# Whole organ and islet of Langerhans dosimetry for calculation of absorbed doses resulting from imaging with radiolabeled exendin

**DOI:** 10.1038/srep39800

**Published:** 2017-01-09

**Authors:** Inge van der Kroon, Wietske Woliner-van der Weg, Maarten Brom, Lieke Joosten, Cathelijne Frielink, Mark W. Konijnenberg, Eric P. Visser, Martin Gotthardt

**Affiliations:** 1 Department of Radiology and Nuclear Medicine, Radboud University Medical Center, Nijmegen, Netherlands; 2 Department of Nuclear Medicine, Erasmus Medical Center, Rotterdam, Netherlands

## Abstract

Radiolabeled exendin is used for non-invasive quantification of beta cells in the islets of Langerhans *in vivo*. High accumulation of radiolabeled exendin in the islets raised concerns about possible radiation-induced damage to these islets in man. In this work, islet absorbed doses resulting from exendin-imaging were calculated by combining whole organ dosimetry with small scale dosimetry for the islets. Our model contains the tissues with high accumulation of radiolabeled exendin: kidneys, pancreas and islets. As input for the model, data from a clinical study (radiolabeled exendin distribution in the human body) and from a preclinical study with Biobreeding Diabetes Prone (BBDP) rats (islet-to-exocrine uptake ratio, beta cell mass) were used. We simulated ^111^In-exendin and ^68^Ga-exendin absorbed doses in patients with differences in gender, islet size, beta cell mass and radiopharmaceutical uptake in the kidneys. In all simulated cases the islet absorbed dose was small, maximum 1.38 mGy for ^68^Ga and 66.0 mGy for ^111^In. The two sources mainly contributing to the islet absorbed dose are the kidneys (33–61%) and the islet self-dose (7.5–57%). In conclusion, all islet absorbed doses are low (<70 mGy), so even repeated imaging will hardly increase the risk on diabetes.

The beta cells in the islets of Langerhans in the pancreas produce insulin and play a key role in maintaining blood glucose levels within a normal range. In diabetes, the glucose homeostasis is disturbed by different underlying pathologies, resulting in insufficient beta cell function and/or mass. Until recently, the relation between beta cell mass (BCM) and beta cell function could not be investigated *in vivo* in humans due to the lack of a method to measure the BCM non-invasively.

Therefore, there is a major need for non-invasive determination of BCM in patients with diabetes^1^, and people at risk of diabetes. Such a method could also provide more insight in the relation between BCM and function in healthy volunteers as compared to a population at risk or with diabetes. In the last couple of years various radiolabeled tracers have been developed for BCM quantification by PET and SPECT and evaluated in preclinical studies^2^ with the first tracers now being tested in clinical trials^3^,^4^,^5^. One promising tracer for beta cell quantification is radiolabeled exendin, which binds specifically to the beta cells^6^ via the glucagon-like peptide-1 (GLP-1) receptor and is internalized upon receptor binding^3^. This internalization and subsequent “metabolic trapping” of the tracer, labeled with a radiometal^3^, leads to high target-to-background ratios.

The beta cells are the main cell type in the islets of Langerhans (~80% of the cells)^7^, and are assumed to be homogenously distributed within the islets in human^8^. Besides beta cells, the islets of Langerhans consist of alpha, delta and pp cells. The islets of Langerhans are approximately spherical structures with diameters between 50 and 400 μm. The different cells types within the islets are of vital importance for maintaining glucose homeostasis and the accumulation of radioactivity leads to concerns about possible radiation-induced damage to the islets, especially after repeated radiotracer administration. To ensure imaging without damaging the islets, a method to calculate the islet absorbed dose is required.

Until now, no suitable model has been available to calculate absorbed dose in the islets. Some attempts have been made using whole organ-based dosimetry to calculate average whole body and pancreas absorbed doses when using radiolabeled exendin^9^,^10^,^11^,^12^. Mikkola *et al*.^9^ used the rat biodistribution of NODAGA-exendin, labeled with ^64^Cu or with ^68^Ga to estimate human whole body absorbed doses with OLINDA/EXM^13^. In a similar way, Wild *et al*.^10^ used the biodistribution in Rip1Tag2 mice for three different exendin radiopeptides (labeled with ^68^Ga, ^111^In or ^99m^Tc) to calculate the total body and pancreas dose. Recently, Selvaraju *et al*.^12^ calculated organ absorbed doses after administration of ^68^Ga-exendin in different animal species and humans. In all studies the pancreas absorbed dose was calculated assuming a homogenous radioactivity distribution in the pancreas. However, only 1–2% of the pancreatic tissue consists of islets and the activity concentration in an islet is much higher than in exocrine tissue^3^,^9^. Therefore, the absorbed dose in the islets is expected to be higher than in the exocrine tissue, which is not reflected by the models used so far, although acknowledged by Velikyan *et al*.^11^. Therefore, the islets should be included as a separate compartment in the islet absorbed dose calculation.

In this work, whole organ and small scale (μm-level) dosimetry was combined to calculate the islet absorbed dose after administration of radiolabeled exendin. In a previous clinical study, high accumulation of radiolabeled exendin in the pancreas and the kidneys was observed, while the radioactivity concentration in the remainder of the body was negligible^3^. Therefore, the kidneys and pancreas were used as source organs in the organ level dosimetry part of the model and the remainder of the body was not included. To complete the model, small scale dosimetry of the islets was included.

The islet self-dose was calculated using Monte Carlo based small scale dosimetry using the MCNP code^14^, whereas the absorbed doses due to kidneys and pancreas were calculated using S-values obtained from the OLINDA/EXM^11^ software. OLINDA/EXM is a software program which can be used to obtain organ S-values or calculate organ absorbed doses after administration of radiopharmaceuticals. An S-value describes the contribution of the activity in one organ (source) to the dose in another organ (target). Accurate dose calculations require detailed information about the tracer distribution not only for different organs but also within specific organs. Therefore, not only organ based data on tracer distribution from a clinical study but also specific information on the tracer uptake in exocrine tissue and islets within the pancreas retrieved from preclinical data, were used as input for the model.

Finally, the model was applied to estimate islet absorbed doses and to investigate the effect of different radionuclides (^111^In or ^68^Ga) and different biological factors (male or female, low or high kidney uptake, small or large islets, healthy or diabetic subjects) on the absorbed dose in the islets.

## Methods

### Dosimetry model

**Figure 1:**
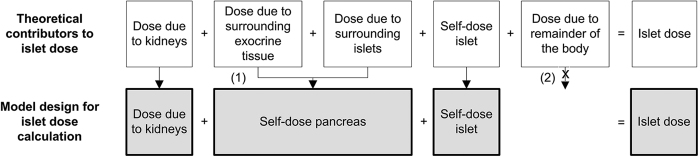
The different sources that contribute to the islet absorbed dose and the assumptions made in the model. Under the assumption of homogenous distribution of the islets through the pancreas, the contribution of the surrounding islets and exocrine pancreas is combined in the self-dose of the pancreas (1). Based on previous observations the activity in the remainder of the body is neglected (2).

The absorbed dose in one islet is the sum of absorbed doses originating from different sources. When source and target are the same, the resulting absorbed dose is called self-dose. Figure 1 shows the sources that contribute to the islet absorbed dose and the assumptions made in our model. We assume that (I) all islets are homogeneously distributed through the pancreas and that (II) the surrounding islets and the exocrine tissue contribute to the islet absorbed dose as one source with a uniform distribution. This is defined as the self-dose of the pancreas.

Using these assumptions, the islet absorbed dose (*D*_*islet*_) is given by:


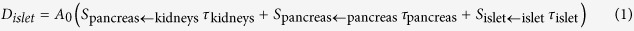


where S-values describe the contribution of activity of one tissue (source) to the absorbed dose in another tissue (target), *τ* is the time integrated activity coefficient and *A*_0_ the administered activity. For the use of S-values, it is assumed that activity is homogeneously distributed in the source and the absorbed dose is uniform in each target organ^12^. This also holds for the calculation of the islet self-dose, where the islets are considered as spheres of homogeneously distributed activity. The total islet absorbed dose is slightly overestimated, since it is a combination of the islet self-dose and the pancreas self-dose, as the islet for which the islet absorbed dose is calculated is also part of the pancreas.

Model calculations were performed using an administered activity of 150 MBq for ^111^In, and 75 MBq for ^68^Ga (because of the higher sensitivity of PET imaging with ^68^Ga compared to SPECT imaging with ^111^In).

Each following section describes the contribution of one source (kidneys, pancreas and islet) to the islet absorbed dose. For each source we describe in which way S-values and time integrated activity coefficients were obtained and which assumptions were made.

### Kidneys

The average absorbed dose in the islets originating from the kidneys is equal to the average dose to the pancreas originating from the kidneys under the assumption of equal absorption of photons, in both islets and exocrine tissue, the absorption of electrons is neglected due to their short range in tissue. Using this assumption, the islet absorbed dose due to the activity in the kidneys can be estimated using the S-values for the pancreas absorbed dose due to the kidneys (S_pancreas←kidneys_). S-values for an adult male and an adult female, for ^111^In and ^68^Ga (see supplementary material, Table 1) were taken from OLINDA/EXM, and are based on Monte Carlo simulations^15^,^16^.

The time integrated activity coefficient of the kidneys was based on planar scintigraphic images, both anterior and posterior from a previous clinical study (clinical trial number: NCT01825148). The protocol was approved by the Institutional Review Board of the Radboud University Medical Center, written informed consent was obtained from all participants and the study was carried out in accordance with the approved guidelines. In the present dosimetry study, scintigrams of five healthy volunteers (2 male, 3 female, average age: 33.2 ± 13.6 years) acquired 0, 4, 24, 48, 96 and 168 h after injection of 150 MBq ^111^In-exendin-4 were used for calculation of the time integrated activity coefficient of the kidneys for each volunteer. The amount of activity in the kidneys at the different time points and the time integrated activity coefficients for ^111^In were calculated with SPRIND-software^17^. For calculation of the ^68^Ga time integrated activity coefficients, also the ^111^In scintigrams were used, but the activity in the kidneys was corrected for the shorter half-life, assuming similar biological behavior for ^111^In-exendin and ^68^Ga-exendin.

In the islet dosimetry model the average time integrated activity coefficient of these five volunteers was used as input. For simulation of humans with “high” or “low” kidney uptake, the maximum and minimum time integrated activity coefficient of these five volunteers was used, respectively.

### Self- dose pancreas

Within the pancreas, the activity in the exocrine part and in islets contributes to the islet absorbed dose. The S-values for the pancreas self-dose (S_pancreas←pancreas_) were also taken from OLINDA/EXM for an adult male and adult female, for ^111^In and ^68^Ga. (see supplementary material, Table 1).

Since no human data is available on the specific accumulation of ^111^In-exendin in islets and in exocrine tissue an alternative approach using preclinical data was set up to calculate the contribution of the exocrine pancreas and the contribution of the islets to the islet absorbed dose. The animal experiments were approved by the animal welfare committee of the Radboud University Nijmegen and the study was carried out in accordance with the approved guidelines.

Nine female Biobreeding Diabetes Prone (BBDP) rats (5–7 weeks old) (Biomedical Research Models, Worchester, MA, USA), were euthanized two hours post injection (p.i.) of 18.5 ± 1.78 MBq ^111^In-exendin (peptide dose 20 pmol). Pancreata were dissected and activity in the pancreas for each rat was measured in a gamma counter (Wallac 1480 wizard, Perkin Elmer, Waltham, MA, USA), 2 weeks p.i. The pancreata were fixed in formalin, embedded in paraffin and sliced into 4 μm sections for autoradiography and histological evaluation.

**Figure 2:**
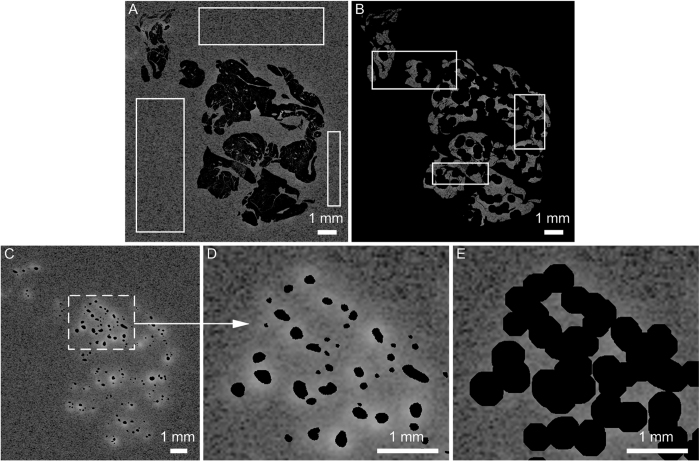
The autoradiography image of supplementary Figure 1 with respectively masks (in black) of: (**A**) pancreatic tissue, (**B**) dilated islets and background and (**C**) islets. (**D**) is a magnification of (**C**) and demonstrates the projection of islet activity outside the islets, so called spill-over. (**E**) visualizes how dilation of the delineated islets (black mask) enables inclusion of all activity originating from the islets, as no spill-over is visible.

For autoradiography, a phosphor imaging plate was exposed to tissue sections of the pancreas (n=3 per rat) for 7 days and developed using a radioluminography laser imager (Fuji Film BAS 1800 II system, Raytest, Straubenhardt, Germany), exported from AIDA software (Raytest) and further processed in MATLAB (version R2011a, The MathWorks, Inc., USA). The same sections were stained for the presence of insulin and the total pancreatic area and the area of the insulin positive islets were determined as described previously^3^. Mask images of these delineated islets and of the total pancreatic area were created and saved. In MATLAB, autoradiography images and their corresponding histological sections were scaled and rotated to ensure optimal registration of the islets in both images. For each tissue section, the islet fraction was calculated by dividing the insulin positive area (islet mask) by the total pancreatic area (tissue mask). Furthermore the average activity outside the tissue, so called background (e.g. due to cosmic radiation) and the average activity in the exocrine tissue were calculated by selecting three regions outside the tissue (Fig. 2A,B). For each tissue section the pixel values in the autoradiography images, representing the activity concentration in the exocrine tissue were corrected for the background.

Figure 2C and D show that in the autoradiography images, activity originating from the islets is also projected in the surrounding of the islets. This spill-over is caused by the distance between the tissue section and the phosphor imaging plate. Delineated islets were dilated (Fig. 2E), to include spill-over originating from the islets in the calculation of the ratio between tracer uptake in the islets and in exocrine tissue. The activity in the dilated part (Fig. 2E) was corrected for exocrine tissue activity, and included in the calculated islet pixel value. Finally, the average uptake ratio per pixel between islet and exocrine tissue (each section separately) can be calculated.

To calculate time integrate activity coefficients for each rat pancreas (exocrine tissue and islets), the fraction of injected activity in each rat pancreas was used. With this time integrated activity coefficient for each rat pancreas, the average uptake ratio per pixel and the islet fraction, the islet and exocrine time integrated activity coefficients can be calculated. Finally these time integrated activity coefficients were translated to human time integrated activity coefficients using the method described by Kirschner *et al*.^18^. A more extensive description of the calculation of the pancreas self-dose is given in the supplementary material.

For calculation of the ^68^Ga time integrated activity coefficients, the activity in the pancreas was corrected for the shorter half-life, assuming similar biological behavior for ^111^In-exendin and ^68^Ga-exendin in the pancreas in rats, and the same translation to human.

### Self-dose islets

Assuming islet sphericity, the sphere model^19^ as used in OLINDA/EXM would be suitable for calculation of the islet self-dose, unfortunately the smallest sphere in OLINDA/EXM (diameter: 0.13 cm)^19^ is much larger than the typical islet size (diameter: 50–400 μm). For calculation of S-values for ^111^In and ^68^Ga in spheres with sizes that cover the range of the islet sizes, new Monte Carlo simulations using the MCNP code^14^ were performed. The methods for these Monte Carlo simulations were described previously^19^, although for the present calculations only MCNP code was used and the comparison with results calculated with the EGS4 code was skipped. For the Auger electrons with energy <1 keV complete local absorption was assumed. For the electrons with energy >1 keV and for photons, Monte Carlo simulations were performed for spheres with diameters of 50, 100, 200 and 400 μm (see supplementary material, Table 2), which covers the typical range of the islet sizes. For each electron, positron and gamma spectrum from the MIRD-07 database for ^68^Ga and ^111^In, 10 million initial particles were used as input, the energy cut-off was set at 1 keV and the ITS 3.0 electron energy loss straggling algorithm was set (DBCN 17j 2)^20^. The density and elemental composition of the pancreas was according to ICRU report 44^21^, the beta cells were modelled according to soft tissue (male) in the same report.

Calculation of the time integrated activity coefficient for the islet was performed as described in self-dose pancreas.

### Use of the model

To demonstrate the application of the model, several examples are described. Islet absorbed doses were calculated for healthy male (I), healthy female (II), male with a high or low kidney uptake (III), male with small or large islets (IV) and diabetic male (V). Islet absorbed doses were calculated for both ^111^In and ^68^Ga (details about the model input for the different examples are given in Table 1).

## Results

Results for the examples specified above, are given in Table 1. Here, we describe the main results.

### The use of ^68^Ga instead of ^111^In will result in a much lower islet absorbed dose

The use of ^68^Ga instead of ^111^In will result in only an islet absorbed dose 0.99 mGy compared to 46.2 mGy (Table 1, example 1). The relative contribution of the islet self-dose to the total islet absorbed dose is smaller for ^68^Ga than for ^111^In (13% versus 39% of the total islet dose), whereas the relative contribution of the kidneys to the islet absorbed dose is in the same range for ^111^In and ^68^Ga (53% and 46%).

### The islet absorbed dose is higher for females than for males

Calculation of the islet absorbed dose for males and females resulted in higher absorbed dose for females (57.6 versus 46.2 mGy for ^111^In and 1.21 versus 0.99 mGy for ^68^Ga (Table 1, example 1 and 2)). Although the absolute absorbed dose is higher for females, the relative contribution of all sources is similar.

### The kidney uptake has a substantial effect on the islet absorbed dose

The islet absorbed dose in a subject with a high kidney uptake was approximately 15–30% higher than in a subject with low kidney uptake (40.0 mGy and 51.1 mGy for ^111^In and 0.92 mGy and 1.07 mGy for ^68^Ga (Table 1, example 3)). This difference in total islet absorbed dose is only a result of the difference in kidney time integrated activity.

### Islet absorbed dose largely depends on islet size

The islet absorbed dose was calculated for both small (50 μm) and large (400 μm) islets. For ^111^In the islet self-dose was 15.4 mGy for islets with a diameter of 50 μm and 37.9 mGy for islets with a diameter of 400 μm (Table 1). The ^68^Ga islet self-dose was 0.07 mGy for 50 μm islets and 0.51 mGy for 400 μm diameter (Table 1, example 4). For the large islets this results in a high relative contribution of the islet self-dose to the total islet absorbed dose (57% for ^111^In and 37% for ^68^Ga). Compared to 50 μm diameter islets, the total islet absorbed dose in 400 μm islets is increased by 52% for ^111^In and by 48% for ^68^Ga.

### The absorbed islet dose is slightly lower in diabetic subjects compared to healthy subjects

To simulate diabetic patients, meaning patients with a decreased BCM, the percentage islets in the pancreas was reduced from 2% to 0.2%. This strongly influenced the self-dose of the pancreas: for ^111^In in male the self-dose drops from 3.68 mGy to 1.51 mGy and for ^68^Ga from 0.40 mGy to 0.16 mGy (Table 1, example 1 and 5). The relative contribution of the whole pancreas to the total islet absorbed dose decreases from 8% in a healthy male to 3% in diabetic male for ^111^In and from 40% to 21% in ^68^Ga.

## Discussion

Up to now, the islet absorbed doses as reported in the literature were based on the mean absorbed dose in the whole pancreas and this could lead to an underestimation of potential islet damage resulting from the use of specific radiolabeled tracer molecules for beta cell imaging such as exendin. We have developed a model to calculate islet absorbed doses. The S-values as implemented in OLINDA/EXM were used to calculate the contribution of the kidneys and pancreas to the islet absorbed dose and small scale dosimetry was used to calculate the islet self-dose. The current model can simulate patients with differences in gender, islet size, percentage of islets in the pancreas and kidney uptake for tailoring the dosimetry to healthy subjects as well as patients with diabetes (thus with a lower percentage of islets).

In all our examples, substantial contribution of the activity in the islet itself to the total islet absorbed dose was found (with the maximum contribution in large islets, for ^111^In 57%, and for ^68^Ga 37%). Therefore, the specific accumulation of activity in the islets should be included in the islet absorbed dose calculation. The larger contribution of the islet self-dose for ^111^In compared to ^68^Ga (both absolute and in terms of percentage) to the total islet absorbed dose can be explained by the longer half-life of ^111^In, but is also a result of the short range of the conversion electrons of ^111^In (Table 2).

The islet self-dose for ^111^In is more than twice as high in 400 μm islets than in 50 μm islets. The increase in islet self-dose is even more pronounced for ^68^Ga, where the islet self-dose is more than 700% higher in large islets than in small islets. This higher islet self-dose in large islets, for both ^111^In and ^68^Ga. results in an increase in total islet absorbed dose of approximately 50%.

In the study of Wild *et al*.^10^ the islets were not included as a separate source and pancreas absorbed doses were calculated for ^111^In-exendin and ^68^Ga-exendin from mice data, resulting in a pancreas dose of 0.7 mGy/MBq for ^111^In-exendin and 0.2 mGy/MBq for ^68^Ga-exendin. Selvaraju *et al*.^12^ also calculated pancreas absorbed doses for ^68^Ga-exendin but in different species and found pancreas absorbed doses between 0.01 and 0.10 mGy/MBq. Inclusion of the islet self-dose in their models would have led to substantially higher islet absorbed doses than their reported average pancreas absorbed doses, because in our study the islet self-dose contributes up to 57% to the total islet absorbed dose.

The higher islet absorbed dose found in human female compared to male is a result of conversion of the rat time integrated activity coefficients to human (total body weight male 70.0 kg, female 56.9 kg) and the different S-values, due to a different distance between the organs in the standard male and female phantoms^15^,^16^.

The five calculated examples showed that the contribution of the activity in the kidneys to the total islet absorbed dose is relevant, ranging from 33% for 400 μm islets in healthy males administered with ^68^Ga exendin, to even 60% in diabetic males administered with ^68^Ga-exendin (100 μm diameter islets). Therefore, reducing kidney uptake (e.g. with gelofusine^24^) is expected to not only increase image quality by increasing the pancreas to kidney activity concentration ratio, but would also lower the islet absorbed dose.

In all examples for which we calculated the islet absorbed dose, the islet absorbed dose was very low (maximum 66.0 mGy for ^111^In and 1.38 mGy for ^68^Ga), hardly increasing the risk on diabetes as a result of radiation^25^. Our calculations also demonstrate that in order to minimize islet absorbed doses, the use of ^68^Ga-exendin is preferred over ^111^In-exendin.

In the future, the model could be refined by separating the pancreas into different compartments. In the current model an average islet absorbed dose is calculated, while the actual islet absorbed dose might for example be higher for islets in close proximity of the kidneys, compared to islets further away from the kidneys. By separating the pancreas into different compartments, islet absorbed doses for separate areas could be calculated. However, to obtain S-values for these compartments in relation to the kidneys and to each other new Monte Carlo simulations are required. Separation of the pancreas in different compartments would also enable simulation of inhomogeneous islet densities within the pancreas, as seen in humans^22^.

In future research the sub-cellular distribution of the radiolabeled exendin should also be taken into account as radiolabeled exendin is internalized in the cells and this could have an effect on the potential cytotoxic effect especially for Auger emitting radionuclides. Several studies on sub-cellular distribution of Auger emitting radionuclides show a significant influence of this sub-cellular distribution on the nucleus absorbed dose^26^,^27^,^28^. Especially if the Auger emitting radionuclide is taken up in the nucleus an increased nucleus absorbed dose is observed with potentially an increased cytotoxic effect on the cells. However, if the radionuclide is not taken up in the nucleus, but only in the cytoplasm and cell surface the average absorbed cell dose would overestimate the nucleus absorbed dose^27^. It is currently not known if radiolabeled exendin is taken up in the cell nucleus. Uptake of the radiolabeled exendin in the nucleus would increase the nucleus absorbed dose for ^111^In-exendin, due to the short range (0.25–16 nm) of the most abundant Auger electrons (8.5 and 350 eV) and thereby the potential cytotoxic effect. However, when there is no uptake in the cell nucleus no enhanced nucleus absorbed dose and therefore cytotoxic effect is expected. To be able to accurately calculate this nucleus absorbed dose, more research on the sub-cellular radiolabeled exendin distribution is necessary.

Here, we have successfully combined human planar images, rat biodistribution data and *ex vivo* autoradiography to calculate the absorbed dose at islet level. The low maximum calculated islet dose (maximum 66 mGy) indicates that even repeated exendin imaging will hardly increase the risk on diabetes^25^. Our data reveal that inclusion of the islet self-dose substantially contributes (up to 57%) to the islet absorbed dose, confirming that the islet self-dose should be included in the dose calculation.

## Additional Information

**How to cite this article**: van der Kroon, I. *et al*. Whole organ and islet of Langerhans dosimetry for calculation of absorbed doses resulting from imaging with radiolabeled exendin. *Sci. Rep.* **7**, 39800; doi: 10.1038/srep39800 (2017).

**Publisher's note:** Springer Nature remains neutral with regard to jurisdictional claims in published maps and institutional affiliations.

## Supplementary Material

Supplementary Data

## Figures and Tables

**Table 1:** Details and results of the examples.

Input	Example I		Example II		Example III				Example IV				Example V	
	^111^In	^68^Ga	Female ^111^In	Female ^68^Ga	High kidney uptake ^111^In	Low kidney uptake ^111^In	High kidney uptake ^68^Ga	Low kidney uptake ^68^Ga	Small islets ^111^In	Large islets ^111^In	Small islets ^68^Ga	Large islets ^68^Ga	Type 1 diabetes ^111^In	Type 1 diabetes ^68^Ga
Gender	M	M	*F*	*F*	M	M	M	M	M	M	M	M	M	M
Radionuclide	^111^In	^68^Ga	^111^In	^68^Ga	^111^In	^111^In	^68^Ga	^68^Ga	^111^In	^111^In	^68^Ga	^68^Ga	^111^In	^68^Ga
Injected activity (MBq)	150	75	150	75	150	150	75	75	150	150	75	75	150	75
Islet diameter (μm)	100	100	100	100	100	100	100	100	*50*	*400*	*50*	*400*	100	100
Time integrated activity coefficient one islet (MBq.h/MBq)	1.34E-8	2.24E-10	1.73E-8	2.90E-10	1.34E-8	1.34E-8	2.24E-10	2.24E-10	1.67E-9	8.56E-7	2.80E-11	1.44E-8	1.34E-8	2.24E-10
Percentage islets in pancreas (%)^22^	2	2	2	2	2	2	2	2	2	2	2	2	*0.2*	*0.2*
Time integrated activity coefficient pancreas (MBq.h/MBq)	6.70E-2	1.10E-3	7.90E-2	1.30E-3	6.70E-2	6.70E-2	1.10E-3	1.10E-3	6.70E-2	6.70E-2	1.10E-3	1.10E-3	2.76E-2	5.00E-4
Time integrated activity coefficient kidneys (MBq.h/MBq)	30.7	0.50	30.7	0.50	*36.9*	*22.9*	*0.58*	*0.42*	30.7	30.7	0.50	0.50	30.7	0.50
Results														
Pancreas absorbed dose due to kidneys (mGy)	24.4	0.46	29.4	0.53	29.3	18.2	0.54	0.39	24.4	24.4	0.46	0.46	24.4	0.46
Self-dose pancreas (mGy)	3.68	0.40	4.76	0.52	3.68	3.68	0.40	0.40	3.68	3.68	0.40	0.40	1.51	0.16
Self-dose islet (mGy)	18.2	0.13	23.5	0.16	18.2	18.2	0.13	0.13	15.4	37.9	0.07	0.51	18.2	0.13
Total islet absorbed dose (mGy)	**46.2**	**0.99**	**57.6**	**1.21**	**51.1**	**40.0**	**1.07**	**0.92**	**43.4**	**66.0**	**0.93**	**1.38**	**44.1**	**0.76**
Specific islet absorbed dose (mGy/MBq)	**0.31**	**0.01**	**0.38**	**0.02**	**0.34**	**0.27**	**0.01**	**0.01**	**0.29**	**0.44**	**0.01**	**0.02**	**0.29**	**0.01**

**Table 2:** Characteristics of ^111^In and ^68^Ga.

	^111^In	^68^Ga
**Half-life**	2.80 days	67.71 minutes
**Type of decay**	Electron capture	β^−^
**Gamma energy (keV)^20^**	171.3 (94%)245.4 (91%)	511 (178%)1077 (3.2%)1883 (0.14%)
**Positron energy (keV)^20^**	—	1899 (max) (yield 0.88)836 (mean)822 (max) (yield 0.01)353 (mean)
**Electron energy (keV)^20^**	Auger Electrons (yield 14.7):0.0085–25.6	(mean energy/decay: 6.75)IC Electrons (yield 0.16):145–245(mean energy/decay: 26.0)—
**Range Soft Tissue**	Auger Electrons:0.25 nm–13.6 μm^23^IC Electrons:205–622 μm^23^	Positron:8.9 mm (max)
