# Supplementary material for: Whole organ and islet of Langerhans dosimetry for calculation of absorbed doses resulting from imaging with radiolabeled exendin
Source: Sci Rep. 2017 Jan 9;7:39800. doi: 10.1038/srep39800 (PMC5220322; doi:10.1038/srep39800)
Supplement: Supplementary Data [file srep39800-s1.pdf]

Whole organ and islet of Langerhans dosimetry for calculation of absorbed doses resulting from imaging with radiolabeled exendin

Inge van der Kroon[1], Wietske Woliner - van der Weg[1], Maarten Brom[1], Lieke Joosten[1], Cathelijne Frielink[1], Mark W Konijnenberg[2], Eric P Visser[1], Martin Gotthardt[1]*

Supplementary material

S-values

Supplementary tabel 1 – S-values retrieved from OLINDA/EXM and used in the islet dosimetry model (M=male, F=female)

S-values (mGy/MBq.s)	^{111}In M beta	photon	total	F beta	photon	total
$S_{pancreas \leftarrow pancreas}$	5.48E-5	4.63E-5	**1.01E-4**	6.08E-5	5.12E-5	**1.12E-4**
$S_{pancreas \leftarrow kidneys}$	0	1.47E-6	**1.47E-6**	0	1.77E-6	**1.77E-6**

S-values (mGy/MBq.s)	^{68}Ga M beta	photon	total	F beta	photon	total
$S_{pancreas \leftarrow pancreas}$	1.22E-3	8.61E-5	**1.31E-3**	1.35E-3	9.53E-5	**1.45E-3**
$S_{pancreas \leftarrow kidneys}$	0	3.47E-6	**3.47E-6**	0	3.97E-6	**3.97E-6**

Calculation self-dose pancreas

In MATLAB, each autoradiography image was scaled and rotated to its corresponding histological section to ensure optimal registration of the islets in the autoradiography image with the islets in the pancreatic tissue (Supplemental figure 1). The registration of the histological images and the masks of islets and pancreatic tissue with the autoradiography images was performed by two researchers (IK and WW). The registration of the histological and autoradiography images was scored from 1-5 (1 - very poor registration and 5 - excellent registration) by consensus. Only images with a score 3-5 (~80% of the images) were included for further analysis.

For each tissue section, the islet fraction($f_{isl,rat}$) was calculated by dividing the insulin positive area (islet mask) by the total pancreatic area (tissue mask). Subsequently, the average activity outside the tissue, so called background and the average activity in the exocrine tissue were calculated by selecting three regions outside the tissue and three regions in the exocrine tissue, respectively.

For each tissue section the pixel values in the autoradiography images, representing the activity concentration in the exocrine tissue were corrected for the background:

$$A_{exo\ rat/pix} = A_{exo,u\ rat/pix} - A_{bg\ rat/pix} \quad (1)$$

Where $A_{bg\ rat/pix}$ is the average background pixel value per section, $A_{exo,u\ rat/pix}$ the uncorrected average pixel value in the exocrine tissue and $A_{exo\ rat/pix}$ the corrected average pixel value in the exocrine tissue.

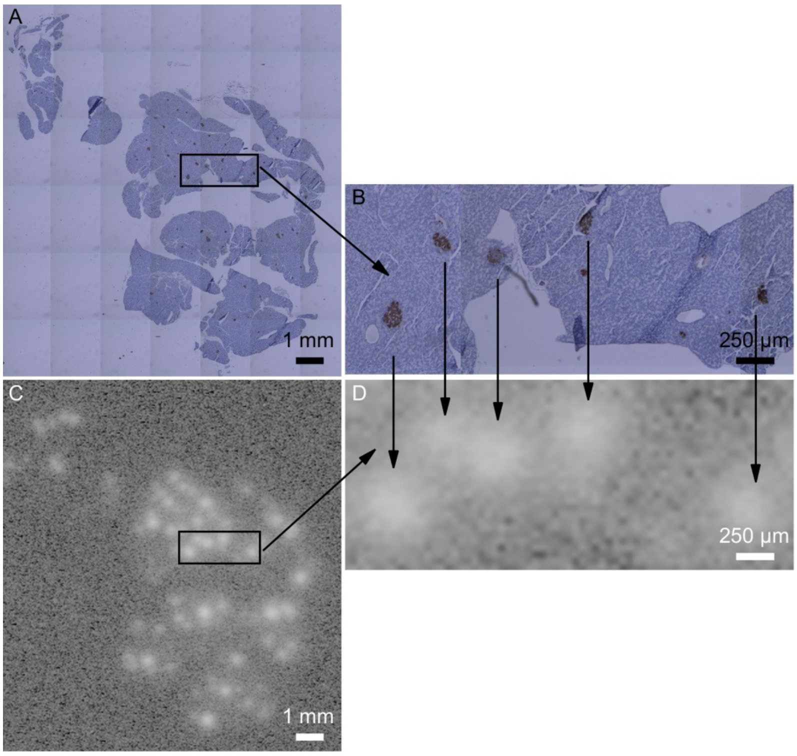

Supplementary figure 1 – An example which shows the correlation of the position of the islets, immunohistochemically stained for insulin in brown (A and B), with the accumulation of ^{111}In-exendin in islets visualized by autoradiography (C and D). B and D are magnifications of the areas indicated by the rectangles in A and C.

In the autoradiography images the activity originating from the islets is also projected in the surrounding of the islets (spill-over). Delineated islets were dilated by 20 pixels on all sides, to include spill-over originating from the islets in the calculation of the ratio between tracer uptake in the islets and in exocrine tissue. The activity in the dilated part (rim) was corrected for exocrine tissue activity, and included in the calculated islet pixel value ($A_{isl\ rat/pix}$):

$$A_{isl\ rat/pix} = A_{isl,u\ rat/pix} + \frac{Area_{rim,rat}\left(A_{rim,u\ rat/pix} - A_{exo,u\ rat/pix}\right)}{Area_{isl,rat}} - A_{bg\ rat/pix} \quad (2)$$

Where $A_{isl,u\ rat/pix}$ is the uncorrected average pixel value in the islet, $Area_{rim,rat}$ is the number of pixels in the dilation area surrounding the islets, $A_{rim,u\ rat/pix}$ is the uncorrected average pixel value in the dilation area, $Area_{isl,rat}$ is the number of pixels in the non-dilated islet area. Finally, the average uptake ratio per pixel between islet and exocrine tissue (each section separately) can be calculated:

$$R_{isl/exo,rat} = \frac{A_{isl\ rat/pix}}{A_{exo\ rat/pix}} \quad (3)$$

For calculation of the time integrated activity coefficients we assumed that immediately after injection the activity in the exocrine pancreatic tissue and in the islets is instantaneously distributed to its final position and from this moment decreases according to physical decay, with no biological clearance or redistribution between islets and exocrine tissue.

Time integrated activity coefficients for each rat pancreas (exocrine tissue and islets) were calculated. The fraction of the injected activity in each rat pancreas ($f_{ID,panc\ rat}$), determined by dividing the pancreatic uptake (extrapolated to t=0 h from the biodistribution data) by the administered activity, was used to calculate the time integrated activity coefficient for each rat pancreas:

$$\tau_{panc\,rat} = f_{ID,panc\,rat} \cdot \int_0^\infty e^{-\lambda t} dt = f_{ID,panc\,rat} \cdot \frac{1}{\lambda} \quad (4)$$

Where λ is the physical decay constant.

The known pancreas weights (0.37-0.67 g) were converted to pancreas volumes ($V_{panc,rat}$) by assuming a pancreas tissue density of 1.079 g/cm^3 [1]. In the autoradiography images the signal in one pixel originates from a tissue section of 4 µm thick leading to a voxel volume, V_{vox} of 50x50x4 µm^3. The time integrated activity coefficient per voxel ($\tau_{panc\,rat/vox}$) for each rat is given by:

$$\tau_{panc\,rat/vox} = \frac{\tau_{panc\,rat}}{\frac{V_{panc,rat}}{V_{vox}}} \quad (5)$$

$\tau_{panc\,rat/vox}$ can be written as the weighted sum of the exocrine and islet time integrated activity coefficients per voxel ($\tau_{exo\,rat/vox}$ and $\tau_{isl\,rat/vox}$, respectively):

$$\tau_{panc\,rat/vox} = (1 - f_{isl,rat}) \cdot \tau_{exo\,rat/vox} + f_{isl,rat} \cdot \tau_{isl\,rat/vox} \quad (6)$$

Under the earlier assumption of no biological clearance and no redistribution of activity between islets and exocrine tissue, equation (3) leads to:

$$\tau_{isl\,rat/pix} = R_{isl/exo,rat} \tau_{exo\,rat/pix} \quad (7)$$

Therefore, equation (6) can be rewritten as:

$$\tau_{panc\,rat/vox} = (1 - f_{isl,rat}) \cdot \tau_{exo\,rat/vox} + R_{isl/exo,rat} \cdot f_{isl,rat} \cdot \tau_{exo\,rat/vox} \quad (8)$$

$$\tau_{exo\,rat/vox} = \frac{\tau_{panc\,rat/vox}}{f_{isl,rat} \cdot R_{isl/exo,rat} + (1 - f_{isl,rat})} \quad (9)$$

To get the $\tau_{isl\,rat/vox}$ equation (6) is rewritten as:

$$\tau_{isl\,rat/vox} = \frac{\tau_{panc\,rat/vox} - \tau_{exo\,rat/vox} \cdot (1 - f_{isl,rat})}{f_{isl,rat}} \quad (10)$$

The time integrated activity coefficients per voxel for both exocrine tissue and islets for each rat were translated to humans with the following equation:

$$\tau_{exo\,human/vox} = \tau_{exo\,rat/vox} \cdot (\frac{W_{TB,rat}}{W_{TB,human}}) \quad (11)$$

$$\tau_{isl\,human/vox} = \tau_{isl\,rat/vox} \cdot (\frac{W_{TB,rat}}{W_{TB,human}}) \quad (12)$$

With for each rat $W_{TB,rat}$, the total body weight of the rat (0.10 - 0.15 kg), and $W_{TB,human}$, the total body weight of the human (male 70.0 kg, female 56.9 kg, as used for calculations in OLINDA/EXM).

Subsequently the time integrated activity coefficient per voxel for the human pancreas is calculated:

$$\tau_{panc\,human/vox} = (1 - f_{isl,human}) \cdot \tau_{exo\,human/vox} + f_{isl,human} \cdot \tau_{isl\,human/vox} \quad (13)$$

Where $f_{isl,human}$ equals the volume fraction of islets in the human pancreas.

The time integrated activity coefficient per voxel for the human pancreas is multiplied by the number of voxels in the pancreas to get the time integrated activity coefficient of the human pancreas:

$$\tau_{panc\ human} = \tau_{panc\ human/vox} \cdot \frac{V_{panc,human}}{V_{vox}} \qquad (14)$$

with $V_{panc,human}$ the volume of the pancreas (which was calculated with the pancreas weight and with the same assumed tissue density of 1.079 g/cm^3 [1] as in rat.

Results Monte Carlo simulations for small spheres

Supplementary table 2 – Results of small sphere Monte Carlo simulations that were included in the model.

	^{111}In			^{68}Ga		
islet diameter (µm)	S-value (mGy/MBq.s) electron	photon	total	S-value (mGy/MBq.s) beta	photon	total
**50**	16847	163	17009	8826	5.5	8832
**100**	2476	38	2515	2089	2.7	2092
**200**	413.9	8.5	422.4	516.5	1.2	517.7
**400**	80.1	1.9	82.0	131.3	0.5	131.8

1. Eckhard, M., Brendel, M. D., Brandhorst, D., Brandhorst, H. & Bretzel, R. G. Can the density of native pancreatic tissue slices predict human islet isolation and purification outcome? *Transplantation proceedings* 36, 2845-2848, doi:10.1016/j.transproceed.2004.09.077 (2004).
